# Using Serum Metabolomics to Predict Development of Anti-drug Antibodies in Multiple Sclerosis Patients Treated With IFNβ

**DOI:** 10.3389/fimmu.2020.01527

**Published:** 2020-07-17

**Authors:** Kirsty E. Waddington, Artemis Papadaki, Leda Coelewij, Marsilio Adriani, Petra Nytrova, Eva Kubala Havrdova, Anna Fogdell-Hahn, Rachel Farrell, Pierre Dönnes, Inés Pineda-Torra, Elizabeth C. Jury

**Affiliations:** ^1^Centre for Rheumatology, University College London, London, United Kingdom; ^2^Centre for Cardiometabolic and Vascular Medicine, University College London, London, United Kingdom; ^3^Department of Neurology and Centre of Clinical Neuroscience, General University Hospital and First Faculty of Medicine, Charles University in Prague, Prague, Czechia; ^4^Department of Clinical Neuroscience, Center for Molecular Medicine (CMM), Karolinska Institutet, Karolinska University Hospital, Huddinge, Sweden; ^5^Department of Neuroinflammation, University College London, Institute of Neurology and National Hospital of Neurology and Neurosurgery, London, United Kingdom; ^6^Scicross AB, Skövde, Sweden

**Keywords:** immunogenicity, anti-drug antibodies, multiple sclerosis, metabolomics, cholesterol, machine learning

## Abstract

**Background:** Neutralizing anti-drug antibodies (ADA) can greatly reduce the efficacy of biopharmaceuticals used to treat patients with multiple sclerosis (MS). However, the biological factors pre-disposing an individual to develop ADA are poorly characterized. Thus, there is an unmet clinical need for biomarkers to predict the development of immunogenicity, and subsequent treatment failure. Up to 35% of MS patients treated with beta interferons (IFNβ) develop ADA. Here we use machine learning to predict immunogenicity against IFNβ utilizing serum metabolomics data.

**Methods:** Serum samples were collected from 89 MS patients as part of the ABIRISK consortium—a multi-center prospective study of ADA development. Metabolites and ADA were quantified prior to and after IFNβ treatment. Thirty patients became ADA positive during the first year of treatment (ADA+). We tested the efficacy of six binary classification models using 10-fold cross validation; k-nearest neighbors, decision tree, random forest, support vector machine and lasso (Least Absolute Shrinkage and Selection Operator) logistic regression with and without interactions.

**Results:** We were able to predict future immunogenicity from baseline metabolomics data. Lasso logistic regression with/without interactions and support vector machines were the most successful at identifying ADA+ or ADA– cases, respectively. Furthermore, patients who become ADA+ had a distinct metabolic response to IFNβ in the first 3 months, with 29 differentially regulated metabolites. Machine learning algorithms could also predict ADA status based on metabolite concentrations at 3 months. Lasso logistic regressions had the greatest proportion of correct classifications [F1 score (accuracy measure) = 0.808, specificity = 0.913]. Finally, we hypothesized that serum lipids could contribute to ADA development by altering immune-cell lipid rafts. This was supported by experimental evidence demonstrating that, prior to IFNβ exposure, lipid raft-associated lipids were differentially expressed between MS patients who became ADA+ or remained ADA–.

**Conclusion:** Serum metabolites are a promising biomarker for prediction of ADA development in MS patients treated with IFNβ, and could provide novel insight into mechanisms of immunogenicity.

## Introduction

Multiple sclerosis (MS) is a progressive neurological disease driven by a combination of inflammatory and neurodegenerative processes. There is currently no cure, but a variety of disease-modifying therapies are now available (1). Many of these are biopharmaceuticals which can elicit an undesirable immune response (immunogenicity) leading to the production of anti-drug antibodies (ADA). The therapeutic consequences of ADA include accelerated/delayed drug clearance, neutralization of bioactivity, cross-reactivity with the endogenous protein and hypersensitivity reactions. Consequently, ADA can compromise treatment efficacy (2–6) and safety (7), and are a clinically significant problem for the treatment of MS.

Beta interferons (IFNβ) have been used to treat MS for more than 20 years (8), reducing relapse rate by ~33% (9). Although drugs that are more effective are now available, IFNβ is still used first line due to its favorable safety profile. However, depending on the formulation, IFNβ can induce ADA at rates varying from up to 30% with subcutaneous injection of IFNβ-1b (Betaferon/Extavia) or IFNβ-1a (Rebif), <5% with intramuscular injection of IFNβ-1a (Avonex) and <1% for PEGylated IFNβ-1a (Plegridy). The type (IFNβ-1b or −1a), route of injection, dose, and frequency of administration all influence the intrinsic immunogenicity of the drug (10).

Numerous studies have demonstrated that persistent high titers of neutralizing antibodies (nAbs) can significantly reduce and even negate the therapeutic benefit of IFNβ treatment (11). At the cellular level, IFN activity can be inferred from the induction of IFN-response genes such as MXA, and nADA have been shown to inhibit MXA induction in a titer-dependent manner (12). The clinical relevance of low nAbs titers and binding antibodies (bAbs) is less clear, but could include immune complex formation and complement activation (13) and increased IFNβ efficacy by lengthening its half-life (14).

It can be difficult to detect loss of efficacy because disease activity is infrequent and can be asymptomatic, and time spent on an ineffective treatment places patients at risk of accruing irreversible neurological damage. Therefore, it is highly desirable to identify patients at high risk of developing immunogenicity prior to therapeutic intervention so that their treatment strategy can be tailored accordingly (15).

However, our understanding of the biological parameters that contribute to an individual's risk of ADA development remains limited. To date, a small number of genetic and immunological parameters have been associated with ADA risk, including human leukocyte antigen (HLA) class II alleles (16), HLA and non-HLA associated single nucleotide polymorphisms (17), and NOTCH2 expression on monocytes (18). Thus, there remains an unmet demand for a predictive biomarker of immunogenicity against IFNβ, and a better understanding of the mechanisms underpinning ADA development is required.

In recent years machine learning (ML) approaches have been applied to clinical problems in MS, including computer-aided diagnosis, neuroimaging analysis and prediction of disease trajectories (19–21). The majority of models have been based on clinical information, but ML-generated serum lipid signatures have also successfully been used to identify (22) and stratify (23) MS patients. Circulating lipids are dysregulated in MS, and have been associated with disease progression (24–28). Indeed, circulating lipids can profoundly influence immune cell behavior (29–32). However, serum lipids have not previously been studied in the context of immunogenicity.

In the present study, serum metabolites and lipids were quantified using an established nuclear magnetic resonance spectroscopy platform (Nightingale Health). A variety of supervised ML methods were applied, including random forest (RF), support vector machien (SVM) and lasso (least absolute shrinkage and selection operator) logistic regression which have all been proved effective for analysis of metabolomics data (22, 33–36). K-nearest neighbors (kNN) was also included for contrast, as in side by side comparisons it has proved inferior to other algorithms (22, 34). Finally decision trees were also implemented due to the ease of interpretation and visualization, although it is acknowledged they are prone to overfitting. Overall, SVM, RF, and logistic regression with/without interactions could all predict future ADA status at baseline or month 3 with F1 score (a measure of accuracy) > 0.735 and specificity > 0.83. Thus, we present a new approach to personalized prediction of ADA development utilizing a combination of serum metabolites and clinical information.

## Materials and Methods

### Patient Cohort

A prospective cohort of MS patients was recruited across six European countries as part of the Anti-Biopharmaceutical Immunization: prediction and analysis of clinical relevance to minimize the RISK consortium (ABIRISK consortium; www.abirisk.eu/). Patients were diagnosed with relapsing remitting multiple sclerosis (RRMS) or clinically isolated syndrome (CIS) according to the revised McDonald criteria 2010 (37). Ethical approval for this study was obtained from the ethics committee of the University College London Hospitals National Health Service Trust, London, United Kingdom (18/SC/0323 and 15/SW/0109), Medical Ethics Committee of the General University Hospital in Prague (125/12, Evropský grant 1.LF UK-CAGEKID), Ethikkommission der Fakultät für Medizin der Technischen Universität München, München, Germany (project no. 335/13), Ethikkommission Nordwest- und Zentralschweiz, Basel, Switzerland (project no. 305/13), and Ethikkommission der Medizinischen Universität Innsbruck, Innsbruck, Austria (UN2013-0040_LEK). All participants provided written informed consent in accordance with the Declaration of Helsinki.

Demographic and clinical information were also recorded, including sex, age, ethnicity, body mass index (BMI), smoking status, type and dose of IFNβ, and expanded disability status score (EDSS) at baseline and 18 months post treatment (Table 1). Smoking status was categorized as never smoked, quit, or current smoker. Patients were on one of four IFNβ formulations: Avonex, Rebif, Betaferon, or Extavia. These were categorized as follows: intramuscular IFNβ-1a (Avonex), subcutaneous IFNβ-1a (Rebif) and subcutaneous IFNβ-1b (Betaferon/Extavia). The dose and frequency of treatment varied between individuals, therefore dose of IFNβ per week was also calculated (dose per administration x frequency of administration).

**Table 1:** Cohort characteristics.

		**ADA– (*n* = 52)**	**ADA+ (*n* = 30)**	***P*-value**
Sex *n (%)*	Female	37 (71)	19 (63)	0.4635^a^
	Male	15 (29)	11 (37)	
Age years	Mean (SD)	34.6 (9.3)	37.9 (9.8)	0.1265^b^
Ethnicity *n* (%)	Caucasian	52 (100)	30 (100)	n/a
BMI	Median (IQR)	23.7 (6.8)	24.6 (7.2)	0.2941^c^
Smoking *n (%)*	Non-smoker	32 (61.2)	16 (53.3)	0.6007^a^
	Quit smoking	8 (15.4)	4 (13.3)	
	Current smoker	12 (23.1)	10 (33.3)	
Type of IFN *n (%)*	Avonex	21 (40)	0 (0)	<0.0001^a^
	Rebif	27 (52)	9 (30)	
	Betaferon/Extavia	4 (8)	21 (70)	
ADA status	nAbs^+^ bAbs^+^	0 (0)	28 (93.3)	n/a
	nAbs^+^ bAbs^−^	0 (0)	2 (6.67)	
Country *n* (%)	Austria	6 (11.5)	3 (10.0)	n/a
	Czech Republic	27 (51.9)	13 (43.3)	
	Germany	6 (11.5)	7 (23.3)	
	Spain	10 (19.2)	1 (3.3)	
	Sweden	1 (1.9)	2 (6.7)	
	Switzerland	2 (3.8)	4 (13.3)	
EDSS at M0	Median (IQR)	2.0 (1.5)	1.5 (2.0)	0.2198^c^
Change in EDSS	Median (IQR)	0 (0.5)	0 (0.63)	0.4902^c^

*Baseline demographic and clinical characteristics were compared between patients who did or did not develop anti-drug antibodies (ADA) to interferon treatment within 1 year. Statistical comparisons were made using ^a^chi-squared, ^b^un-paired two-tailed t-test or ^c^Mann-Whitney U. bAbs, binding antibodies; BMI, body mass index; EDSS, expanded disability status score; IQR, interquartile range; M0, month 0; nAbs, neutralizing antibodies*.

### Separation of Serum and Peripheral Blood Mononuclear Cells

As part of the ABIRISK consortium standard operating procedures were implemented at all sites. Samples were collected prior to IFNβ treatment (M0), and after 3 (M3) and 12 (M12) months of treatment. Peripheral blood samples were non-fasting.

To separate serum from clotted blood, BD SST vacutainers were allowed to coagulate for at least 30 min before centrifugation at 1,500 g for 10 min at 4°C with full acceleration and brake. Serum was aliquoted into screw-capped cryovials and stored at −20°C or below.

To separate peripheral blood mononuclear cells (PBMCs), whole blood was collected into vacutainers containing sodium heparin, and centrifuged at 400 g for 10 min at room temperature (acceleration 5, brake 3) to separate the plasma fraction. Plasma was decanted and heat inactivated (56°C for at least 35 min) before centrifugation at 2,400 g for 15 min (acceleration 9, brake 9). The remaining blood was diluted 1:1 in Roswell Park Memorial Institute (RPMI) 1640 medium supplemented with L-glutamine (Sigma) and layered onto 15 mL Ficoll-Paque PLUS (GE Healthcare) using SepMate tubes (StemCell Technologies) as per the manufacturer's instructions. Cells were washed twice in cold RPMI and resuspended in heat-inactivated autologous plasma with 10% dimethylsulfoxide (Sigma-Aldrich) at a density of ~1 × 10^7^ cells/mL and cryopreserved in liquid nitrogen until use.

### ADA Detection

Serum was tested for both binding (bAbs) and neutralizing (nAbs) ADA. BAbs were measured with an enzyme-linked immunosorbent assay (ELISA) (38) and nAbs were detected by a cell-based luciferase reporter gene assay (39).

As the test for bAbs is less sensitive than the one for nAbs, patients were classified as ADA positive if they were positive for bAbs and nAbs, or were bAbs- but had a nAbs titer ≥ 320 U/mL within 12 months of starting treatment (40) (Table 1). Patients were considered ADA negative if they were negative for both assays. Patients with missing data or negative for bAbs and with a nAbs titer <320 U/mL were excluded from this analysis (*n* = 7).

### Serum Metabolomics Analysis

Measures of 228 serum biomarkers were acquired with a well-established nuclear magnetic resonance (NMR)-spectroscopy platform (Nightingale Health) (41, 42). These included both absolute concentrations, ratios, and percentages of lipoprotein composition. For this study, we have excluded the percentages from analysis leaving 158 metabolite measures (Supplementary Table 1). Serum lipids measured included apolipoproteins (Apo) and (very) low density ((V)LDL), intermediate density (IDL) and high density (HDL) lipoprotein particles of different sizes ranging from chylomicrons and extremely large (XXL), very large (XL), large (L), medium (M), small (S), and very small (XS).

### Predictive Models

**Figure 1:**
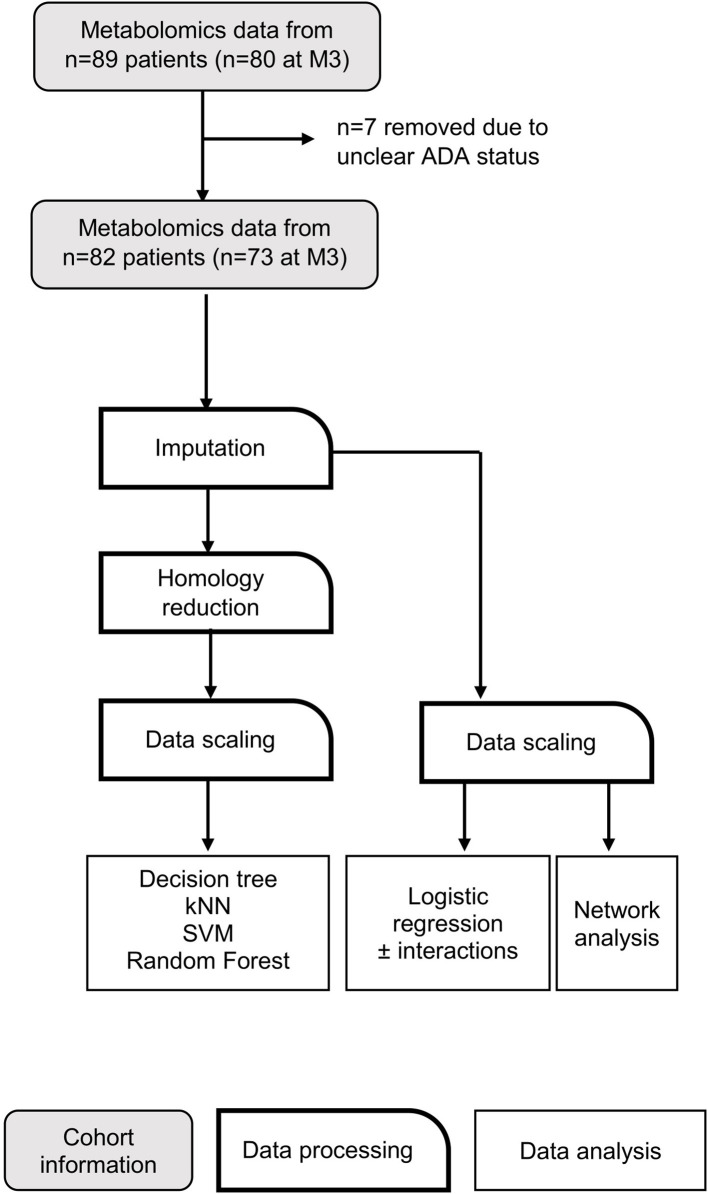
Data analysis workflow. Flow chart depicting data processing steps taken before application of machine learning algorithms. kNN, k nearest neighbors; SVM, support vector machine.

Please consult Figure 1 for a schematic outlining the data analysis pipeline. RStudio (The R Foundation, Vienna, Austria) (43), Orange 3.24.1 (Bioinformatics Lab, University of Ljubljana, Slovenia) (44) and MATLAB (The MathWorks Inc., Natick, USA) were used for machine learning analysis.

Six different supervised learning algorithms were implemented: *k*-nearest neighbors (kNN), support vector machine (SVM), logistic regression with and without interactions, decision trees, and random forest classification. The outcome of the learning algorithms was to predict whether an MS patient is likely to develop ADA in response to IFNβ treatment. Predictive models were generated from metabolite concentrations prior to IFNβ exposure (M0) and after 3 months (M3).

#### Missing Data

Features with >10% missing data were excluded (glutamine and glycerol). Remaining missing values (*n* = 6 M0, *n* = 7 M3) were imputed using *k*-nearest neighbors with *k* = 5.

#### Homology Reduction

Many of the metabolites measured are biologically interdependent, and therefore highly correlated. To reduce homology, if two features had a correlation co-efficient > 0.95 then the feature with the greatest mean absolute correlation with the remaining features was removed (Supplementary File 1). This left 60 metabolites at M0, and 59 metabolites at M3.

#### Data Scaling

Metabolite concentrations were centered on the mean and scaled to the standard deviation.

#### Predictors

The independent variables included in the models were either the full data set (Lasso logistic regression ± interactions and networks) or the homology reduced dataset (60 and 59 metabolites at M0 and M3, respectively), as well as the cohort information (sex, age, BMI, smoking status, country of sample, baseline EDSS, IFNβ type, and dose). Ethnicity was not considered, as all participants were Caucasian. The type of IFNβ was significantly associated with 12 month ADA status, in agreement with other studies (10, 45) (Table 1). Full lists of the predictors contributing to each model are included in Supplementary File 2.

#### kNN

K-nearest neighbors is a non-parametric classification algorithm which assigns the class of an unknown observation based on the class of a number (*k*) of similar observations in the feature space (46). The default value of *k* = 5 was used in this analysis.

#### SVM

Support vector machine is a supervised classification method which creates a hyperplane to optimally separate data into two classes (47). As this data set was not linearly separable, the radial basis function kernel was used. Values for C, epsilon, and gamma were tuned using the R Package e1071 (48). The parameters were set to C = 4.5, epsilon = 0.1, gamma = 0.015 for the M0 model and C = 2, epsilon = 0.2, gamma = 0.01 for the M3 model.

#### Decision Tree

Decision trees are a form of supervised machine learning which outputs a flowchart-like structure, which classifies incidents according to their features. These are built using forms of impurity measures, such as information gain and entropy (49). In an effort to prevent overfitting, decision trees were limited to a depth of 4 and subsets of 5 or less were not split further.

#### Random Forest

Random forest (RF) is a statistical classifier (machine-learning algorithm) that assigns observations into classes (ADA–/+) by creating a set of decision trees, or “forest.” Only a small random sample of predictors are candidates for selection at each node, so the created trees are decorrelated. Ensembling these uncorrelated trees offers a natural way of reducing the variance of the model. Importance was quantified by the Gini index, which represents the total variance across the two classes, the purity of each node and the quality of each split. The optimum number of variables randomly chosen at each node (mtry = 8 and mtry = 11 for M0 and M3, respectively), have been tuned with the function “tuneMTRY” (package “RFmarkerDetector”), with respect to the Out-of-Bag errors. The package “randomForest” function (package “randomForest”) produced RF models which ensemble 1,000,000 trees (50–53).

#### Logistic Regression With/Without Interactions

The least absolute shrinkage and selection operator (lasso) method uses the absolute value of the co-efficient as a penalty to shrink less important features to zero. The strength of shrinkage is determined by tuning the regularization variable lambda (λ). Logistic lasso regression with interactions was conducted with the R package glmnet. All 158 metabolites were included. Categorical predictors were coded as dummy variables with the following treated as the reference class: sex—male, smoking status—never smoked, treatment—Avonex, country—Spain. Age, BMI, baseline EDSS, and dose/week were treated as continuous variables. Ln(λ) was tuned to −2.7 for the logistic regression without interactions (M0 and M3), and −2 (M0) or −2.2 (M3) for the logistic regression + interactions.

#### Model Performance

Ten-fold cross-validation was used to evaluate model performance. The following performance metrics were calculated from the confusion matrices: (1) F1 score–a weighted average of precision (positive predictive value) and recall (sensitivity), (2) specificity–the true negative rate, and (3) classification accuracy (CA)—the proportion of correctly classified cases.

### Logistic Regression

To assess the association of ADA development with NMR metabolomic biomarker data, logistic regressions were performed for each individual serum metabolite, adjusted for sex, age, BMI, smoking status, treatment type and dose, EDSS, and country of sample origin (Supplementary File 3). Standard deviation-scaled odds ratios ±95% confidence intervals were visualized in a forest plot using the R package foresplotNMR (Nightingale Health Ltd) as exemplified in Ahola-Olli et al. (54) (Supplementary Figure 1).

### Network Analysis

Metabolite network diagrams were created with the R package high dimensional undirected graph estimation package [huge (55)]. Graphical lasso (glasso) was used to estimate the sparse inverse covariance matrix, with the stability approach to regularization selection (StARs) (56). The metabolites contributing to each predictive model have been super-imposed onto the network diagrams. Where appropriate, variable importance was determined by ranking mean decrease in Gini (RF) or information gain (SVM). kNN is excluded as this model performed poorly relative to the others.

### Quantification of Cholesterol and Glycosphingolipids by Flow Cytometry

Flow cytometry staining was performed as previously described (57–59). In brief, 1 × 10^6^ PBMCs were stained with Zombie (BioLegend) fixable viability dye for 30 min at 4°C, then labeled with antibodies to surface markers in Brilliant Stain buffer (BD Biosciences) for 30 min at 4°C. Subsequently samples were stained with 25 μg/mL cholera toxin B subunit FITC conjugate (CTB-FITC) (Sigma-Aldrich), fixed for 1 h in 2% paraformaldehyde, and stained for 2 h with 50 μg/mL filipin complex from *Streptomyces filipinensis* (Sigma-Aldrich) before reading the samples on a BD LSRFortessa X-20 cytometer using BD FACSDiva software. Compensation was performed using anti-mouse IgGκ/negative control compensation particles set (BD Biosciences) or OneComp eBeads (ThermoFisher Scientific), with the exception of viability dyes and filipin which were performed with single stained and unstained cells. Data was analyzed using FlowJo (Tree Star).

Antibodies for surface markers: CD45RA-BUV737 (clone HI100, BD Biosciences, 584442) CD27–APC (clone M-T271, BioLegend, 356409), CD4-AF700 (clone OKT4, eBioscience, 56-0048-82), CCR7-BV421 (clone G043H7, BioLegend, 353207), CD69-BV510 (clone FN50, BioLegend, 310936), CD8-BV711 (clone RPA-T8, BioLegend, 301044), CD3-BV785 (clone OKT3, BioLegend, 317330), CD25-PE (clone M-A251, BioLegend, 356104), CD127-PE-Cy7 (clone A019D5, BioLegend, 351320).

### Statistical Testing

Statistical tests were performed in Microsoft Excel and GraphPad Prism version 8.3.0 for Windows (GraphPad Software, San Diego, USA). Data was assessed for normality and analyzed with parametric or non-parametric tests as appropriate. Details of statistical tests are given in the figure legends. *P* < 0.05 were considered statistically significant.

## Results

### Serum Metabolites Can Be Used to Predict Future ADA Development

Metabolites were quantified in serum from MS patients both before IFNβ treatment (month 0–M0) and after 3 months (M3). Patients were classed as ADA positive (ADA+; nAbs+, bAbs+/–) or negative (ADA–; nABs–, bAbs–) based on their ADA status at M12. Several ML models were applied to this data in order to develop a model to predict ADA status (Figure 1). All models were adjusted for sex, age, body mass index (BMI), smoking status, type of IFNβ and weekly dose, country, and baseline expanded disability status score (EDSS).

**Figure 2:**
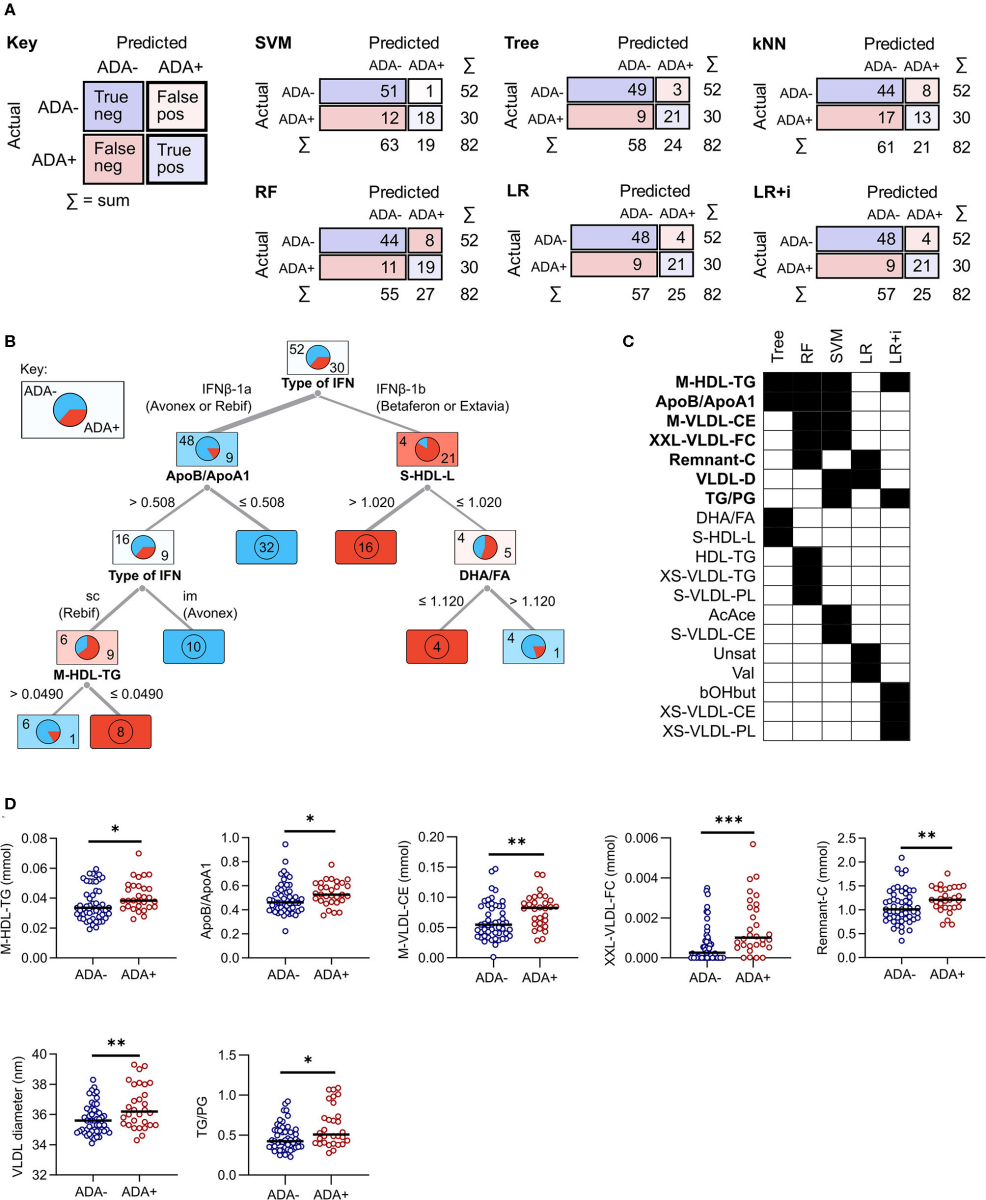
Comparison of predictive model performance at M0. **(A)** The confusion matrix shows the number of correct (blue squares) and incorrect (pink squares) classifications for each model. The sum (Σ) of each row and column is given. The algorithms used were support vector machine (SVM), decision tree (Tree), k-nearest neighbors (kNN), random forest (RF), and lasso logistic regression (LR) with and without interactions (i). **(B)** A graphical representation of the decision tree, where each square shows the proportion of patients who stay ADA negative (top left, blue) or become ADA positive (bottom right, red). The numbers on the branches representcut-off concentrations (mmol) or ratios (ApoB/A1 and TG/PG). **(C,D)** A comparison of the metabolites selected by each machine learning model. For RF and SVM only metabolites within the top 10 predictors are included. Metabolites selected by more than one method are highlighted in bold and shown as dot plots in **(D)**. Line shows the median, and significance was determined by Mann Whitney *U*; *p < 0.03, **p = 0.01, ***p = 0.0003. im, intramuscular; sc, subcutaneous.

At M0 all models were better at predicting ADA– individuals (specificity) than ADA+ (F1 value) (Table 2A). Overall the logistic regression (LR), LR+i (Table 3) and decision tree performed comparably when predicting ADA+ cases, correctly identifying 21 out of 30 (70%) (Figure 2A). On the other hand, the SVM performed better for ADA– cases, with excellent specificity (0.981, Table 2A), only misclassifying one ADA– patient (Figure 2A). Overall the tree had the best performance, with an F1 score of 0.788 (Table 2A, Figure 2B). Seven lipid measures featured in more than one model (Figure 2C), which were all significantly elevated in the patients who went on to develop ADA (ADA+) (Figure 2D). Three of these lipid metabolites (M-VLDL-CE, TG/PG, and XXL-VLDL-FC) represent clusters of highly correlated metabolites, particularly measures of VLDL composition (Supplementary Table 2, Supplementary File 1), indicative of broader differences in metabolite expression. The association between individual metabolites and future ADA status were examined by performing logistic regressions on a per metabolite basis (Supplementary Figure 1, Supplementary File 3). No significant associations were detected demonstrating the importance of accounting for the dependence between metabolites.

**Table 2:** Comparison of predictive model performance.

**Model**	**F1**	**Precision**	**Recall**	**Specificity**	**CA**
**A**					
kNN	0.510	0.619	0.433	0.846	0.695
Tree	0.778	0.875	0.700	0.942	0.854
RF	0.678	0.690	0.667	0.827	0.768
SVM	0.735	0.947	0.600	0.981	0.842
LR	0.764	0.840	0.700	0.923	0.842
LR + i	0.764	0.840	0.700	0.923	0.842
**B**					
kNN	0.571	0.636	0.519	0.826	0.712
Tree	0.741	0.741	0.741	0.848	0.808
RF	0.764	0.750	0.778	0.848	0.822
SVM	0.745	0.792	0.704	0.891	0.822
LR	0.808	0.840	0.778	0.913	0.863
LR + i	0.808	0.840	0.778	0.913	0.863

***(A,B)** Performance statistics for five predictive models based on serum metabolites at M0 **(A)** and M3 **(B)**. The models used were k-nearest neighbors (kNN), decision tree (Tree), random forest (RF), support vector machine (SVM), and logistic regression (LR) with and without interactions (i). The classification accuracy (CA) represents the proportion of correctly identified cases, in contrast to specificity, which is the true negative rate. F1 is the weighted average of the precision and recall (see Methods). Statistics are rounded to 3 decimal places*.

**Table 3:** Predictors for lasso logistic regression with interactions.

**M0 features**	**M3 features**
(Intercept)	(Intercept)
Treatment (IFNβ-1b)	Treatment (IFNβ-1b)
bOHBut: TG/PG	AcAce: EDSS
M-HDL-TG: XS-VLDL-CE	bOHBut: His
TG/PG: XS-VLDL-PL	Lac: Val
BMI: Treatment (IFNβ-1b)	Val: Smoking (Quit)
	VLDL-D: Country (Germany)
	XXL-VLDL-TG: Treatment (IFNβ-1a sc)
	BMI: Treatment (IFNβ-1b)

*List of features selected by the lasso regressions with interactions at month 0 (M0) and M3 (M3). Interacting features are separated by a colon (:). Where predictors are categorical (treatment, smoking status, country) the specific category is shown in brackets. BMI, body mass index; EDSS, expanded disability status score; sc, subcutaneous administration*.

**Figure 3:**
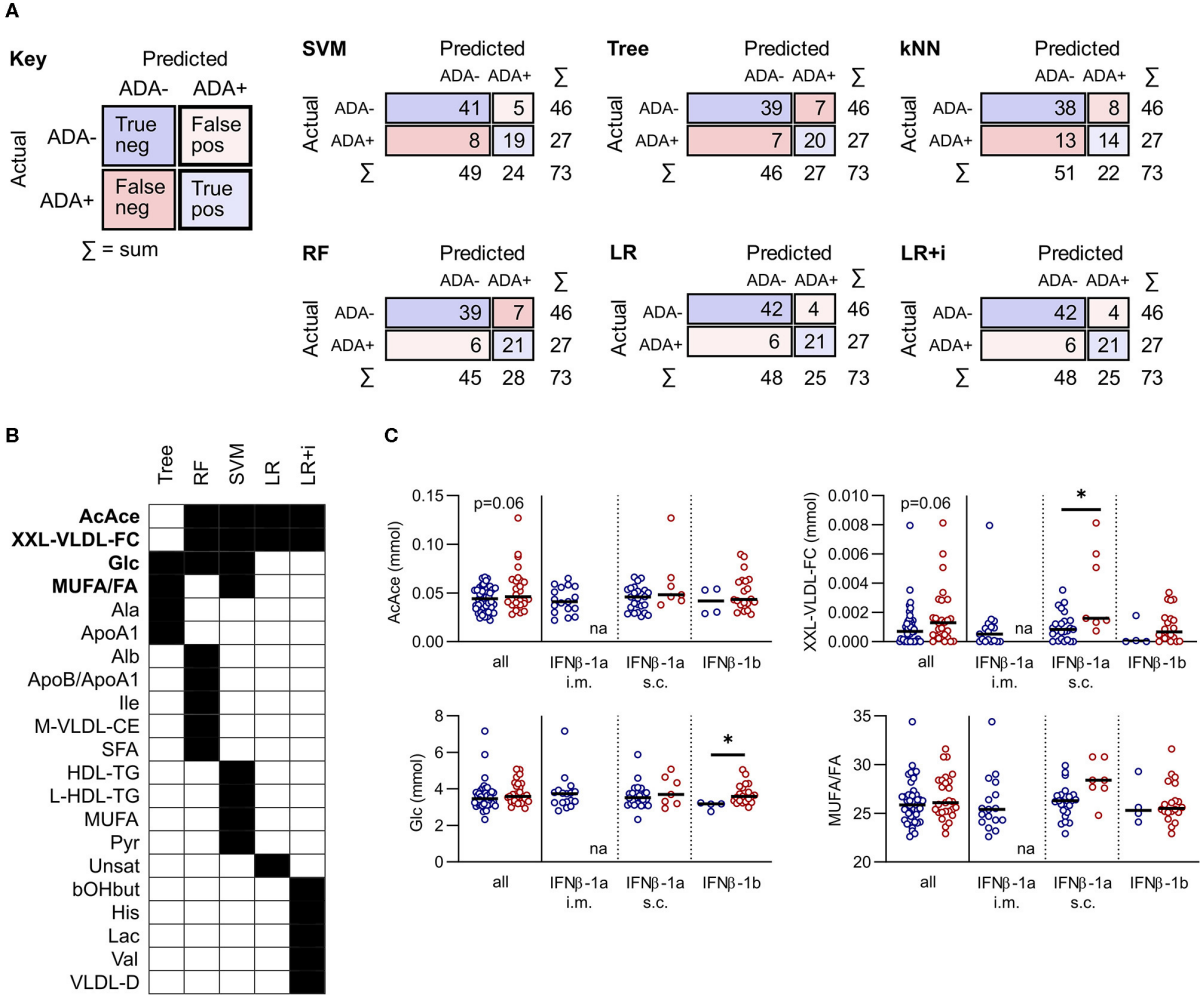
Comparison of predictive model performance at M3. **(A)** Confusion matrices for six predictive models at month 3 (M3): support vector machine (SVM), decision tree (Tree), k-nearest neighbors (kNN), random forest (RF), and lasso logistic regression (LR) with and without interactions (i). **(B,C)** A comparison of the metabolites selected by each machine learning model. For RF and SVM only metabolites within the top 10 predictors are included. Metabolites selected by more than one method are highlighted in bold and shown as dot plots in **(C)**. The dot plots compare ADA– to ADA+ altogether (left), or by stratified by treatment (right). Line shows the median. Statistical significance was determined by the Mann-Whitney U, or the Kruskal-Wallis test followed by Dunn's test for multiple comparisons to compare ADA– and ADA+ within treatment subgroups; *p < 0.05; na, non-applicable.

IFNβ exerts widespread effects on the immune system. Since the response to IFNβ treatment can also influence the development of immunogenicity, similar models were constructed based on serum metabolite concentrations at M3. The best performing models at this time point were again the LR and LR+i (Table 3, Supplementary File 2), which had the highest F1 and specificity values, and lowest total number of misclassifications (Table 2B, Figure 3A). As at M0, all of the models were better at predicting ADA– (specificity) than ADA+ (F1 value) (Table 2B). Four metabolites featured in multiple models (Figure 3B), but in contrast to M0 few of these were differentially expressed when comparing ADA– to ADA+ (Figure 3C, “all”). However, when patients were stratified by treatment type more differences were revealed. Glucose (Glc) levels differed in patients treated with IFNβ-1b, whereas XXL-VLDL-FC was raised in ADA+ patients treated with subcutaneous IFNβ-1a (Figure 3C). The LR+i also selected the interaction between subcutaneous IFNβ-1a and XXL-VLDL-TG (Table 3), which is highly correlated with XXL-VLDL-FC (Supplementary Table 2). Indeed, XXL-VLDL-FC is highly correlated to many other VLDL measures (Supplementary Table 2), 12 of which were also found to be significantly associated with ADA status on a per metabolite basis (Supplementary Figure 1, Supplementary File 3). Only XXL-VLDL-FC was selected by multiple models at both time points (Figures 2C, 3B), with a greater concentration in ADA+ patients (Figures 2D, 3C). This suggests that a cluster of interconnected VLDL lipids may be persistently associated with an increased risk of developing ADA.

The majority of metabolites were not predictive at both time points, suggesting it could be beneficial to implement predictive models both before and after exposure to IFNβ. We examined the longitudinal concordance in predictions for each model (Supplementary Table 3). The logistic regressions generated the same predictions at both timepoints for all but one patient. The decision tree had the highest rate of discordance, particularly in the positive class (38%), coinciding with a reduction in performance at M3. In contrast The RF had a high discordance rate in the ADA– class (21%). This demonstrates that different models have different advantages—some are better at predicting positive cases or negative cases, or are better at M0 or at M3.

### Metabolite Interactions and IFNβ Response Differ Between ADA+ and ADA– Patients

**Figure 4:**
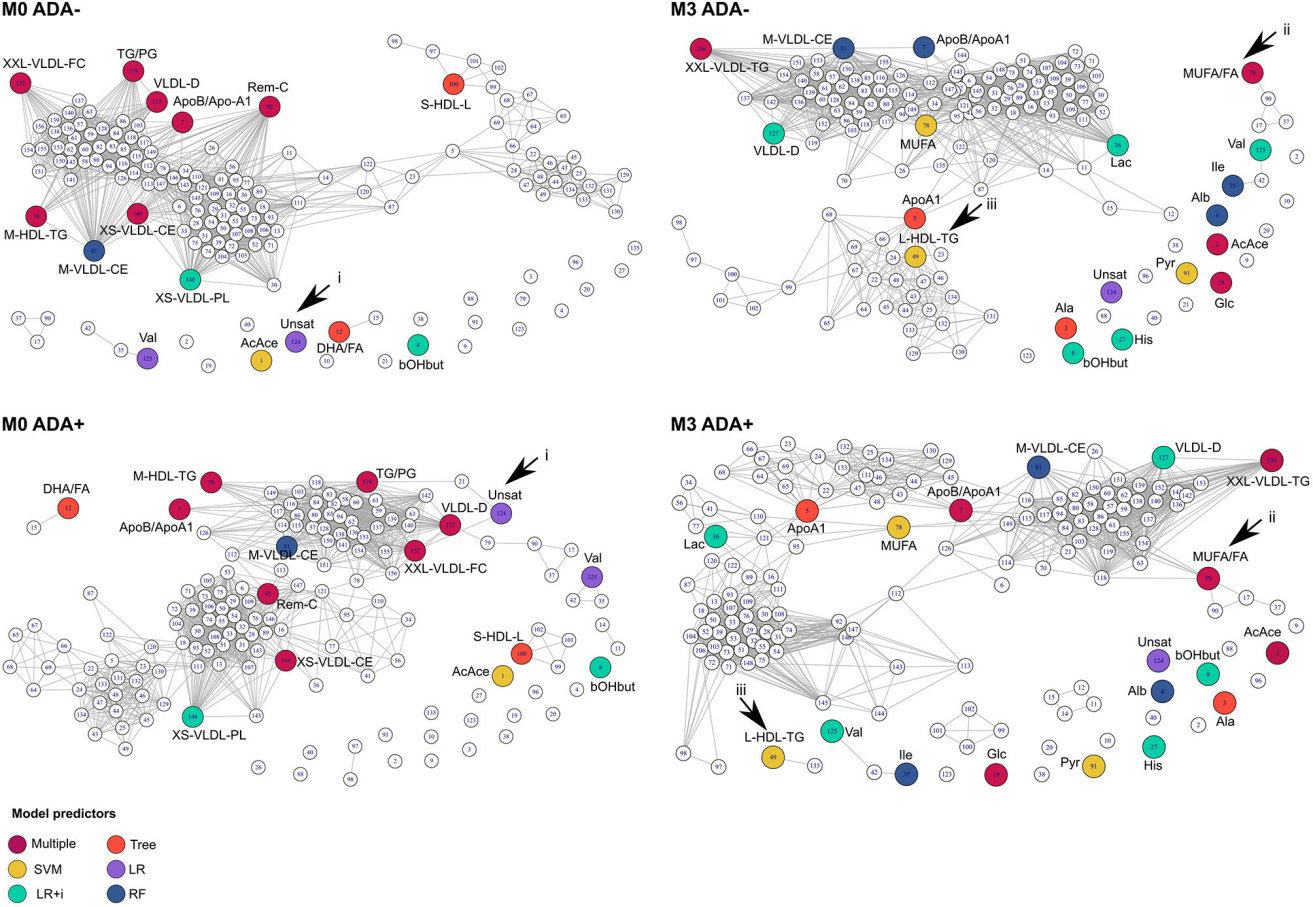
Connections between metabolites are different in ADA– and ADA+ patients. Relationships between metabolites in ADA– and ADA+ patients at baseline (M0) and month 3 (M3) are shown as network diagrams. Colored nodes represent the metabolites contributing to predictive models at each time point (see key “Model predictors”), as described in the methods section. Arrowheads point to key differences that are discussed in the main text.

In addition to examining individual metabolite concentrations we compared metabolite networks in ADA– and ADA+ before and after IFNβ treatment (Figure 4). The metabolite networks were more tightly clustered in ADA– patients at both time-points. A number of metabolites had very different positions depending on ADA status. For instance, Unsat (Figure 4, i) and MUFA/FA ratios (Figure 4, ii) had more connections in ADA+ patients, whereas at M3, L-HDL-TG (Figure 4, iii) lost its relationship with the main metabolite cluster in ADA+ patients.

**Figure 5:**
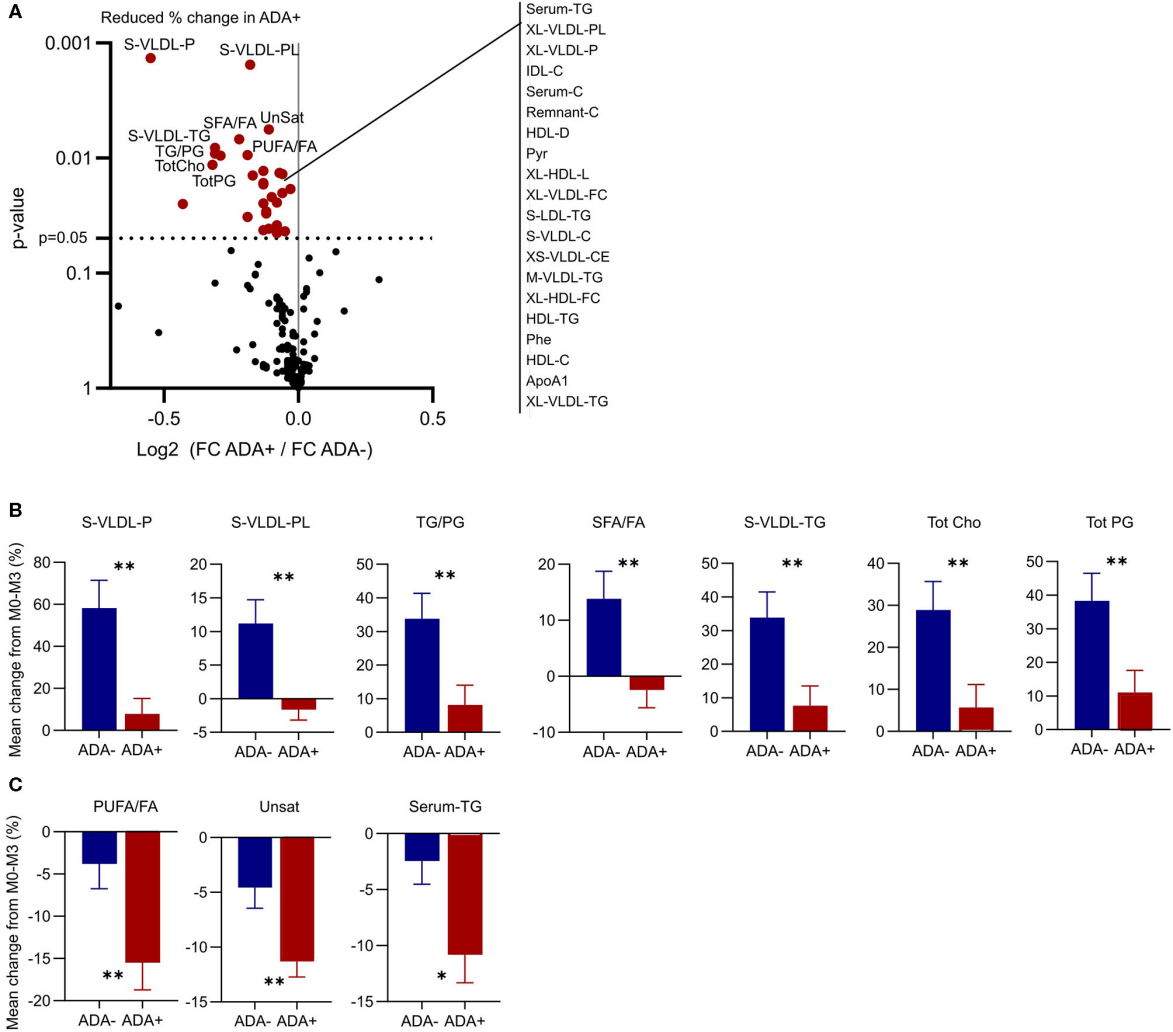
ADA– and ADA+ respond differently to IFN-β treatment. **(A)** Volcano plot to show differences in the metabolic response to IFN-β treatment between ADA+ and ADA–. The 10 metabolites with the most significantly different regulation are labeled, and the remainder with *p* < 0.05 are listed to the right. **(B,C)** The percentage change in the top 10 metabolites in ADA– (blue) and ADA+ (red) are shown as mean + SEM. Some metabolites were increased in ADA–, but not in ADA+ **(B)**. Others were decreased in ADA– and ADA+, but by a greater magnitude in ADA+ **(C)**. Un-paired *t*-test with Welch's correction for unequal variance; *p < 0.05, **p < 0.01.

In both patient groups IFNβ treatment considerably altered the shape of the network (Figure 4, compare M0 vs. M3 in ADA+ and ADA– patients). Therefore, we examined the response to IFNβ in more detail. In total 29 metabolites were differentially regulated between ADA– and ADA+ during the first 3 months of IFNβ treatment (Figure 5A). Some of the metabolite increases induced by IFNβ in ADA– patients were inhibited in ADA+ (Figure 5B). Other metabolites were more suppressed in ADA+ compared to ADA– individuals (Figure 5C). This suggested that IFNβ had an enhanced lipid-lowering effect in ADA+ patients.

### Plasma Membrane Lipid Rafts Are Dysregulated in MS Patients Who Develop ADA

**Figure 6:**
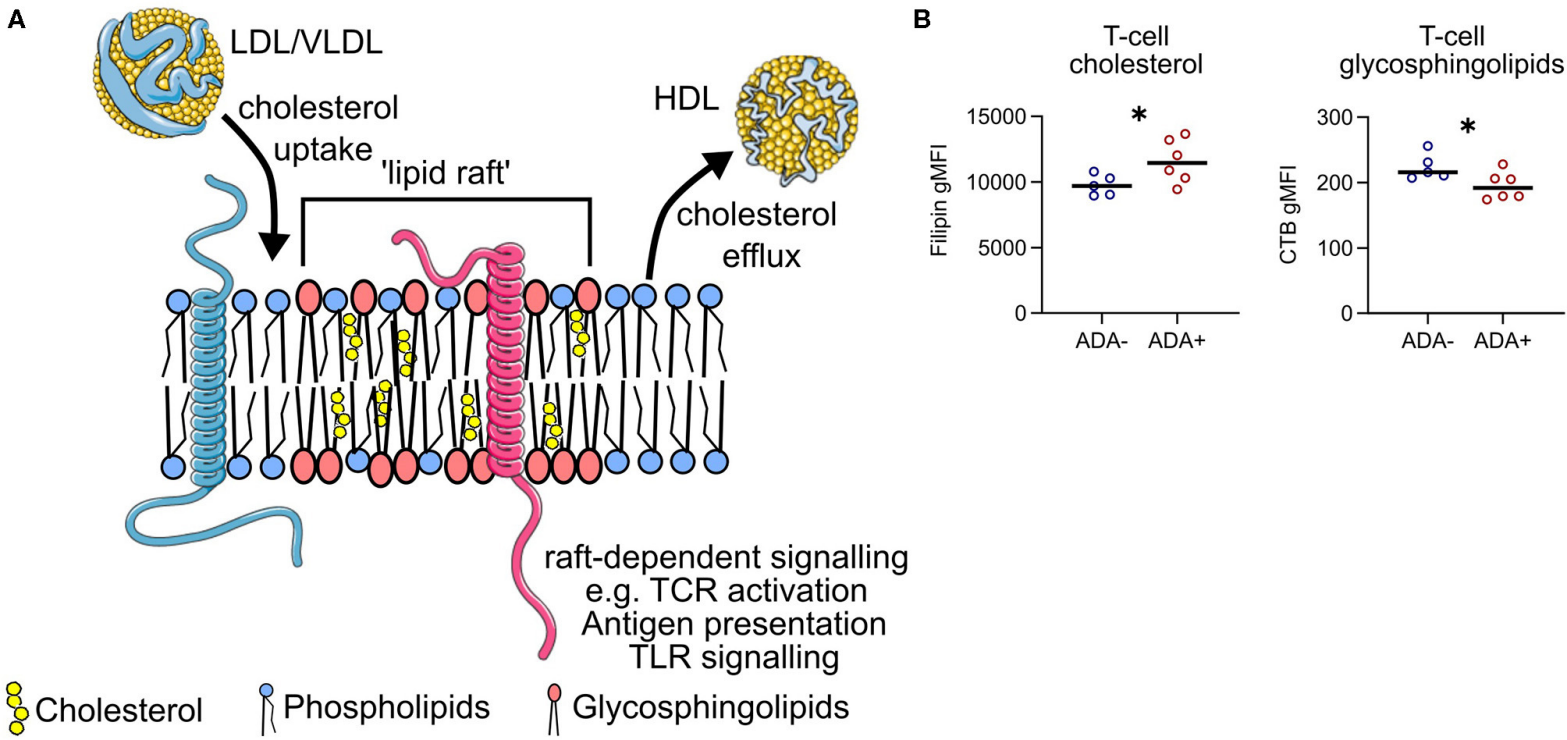
Lipid raft-associated proteins are differentially expressed between ADA– and ADA+. **(A)** Lipoproteins can add or remove cholesterol from the plasma membrane of immune cells. This can alter the composition of “lipid rafts”—membrane microdomains enriched for glycosphingolipids and cholesterol. The tight packing of these lipids generates a region of relative “order” which can selectively attract membrane signaling proteins (e.g., pink protein), whilst excluding others (e.g., blue protein). Examples of raft-dependent signaling include T cell antigen receptor (TCR) signaling, antigen presentation and pro-inflammatory toll-like receptor (TLR) signaling. **(B)** Cholesterol and glycosphingolipid levels were measured in CD4+ T cells from ADA– (*n* = 5) or ADA+ (*n* = 6) multiple sclerosis patients at M0, in five independent experiments. Binding of filipin to cholesterol and cholera-toxin B (CTB) to glycosphingolipids was assessed by flow cytometry. Un-paired two-tailed *t*-test; *p < 0.05.

Serum lipids can modulate immune cell function by altering the composition of plasma membrane lipid rafts; glycosphingolipid and cholesterol enriched microdomains that regulate cell signaling by regulating the lateral mobility of membrane proteins (Figure 6A). Before exposure to IFN (M0) plasma membrane cholesterol was higher and glycosphingolipids were lower in CD4+ T cells isolated from ADA+ patients (Figure 6B). This could suggest that differences in serum lipid composition, for example the observed changes in M-HDL-TG or XXL-VLDL-FC, could generate an immune cell phenotype that predisposes an individual to immunogenicity.

## Discussion

Serum metabolites are attractive candidate biomarkers in MS, and have already been shown to have diagnostic (22, 23, 60) and prognostic (27, 61, 62) potential. Furthermore, they are relatively inexpensive to measure, and a blood-draw is less invasive and time-consuming than a lumbar puncture or MRI scan. We measured serum metabolites at an unprecedented level of detail and, using a combination of ML models, we demonstrated that a subset of serum lipid metabolites could predict ADA development against IFNβ in MS patients.

Future ADA status could be predicted before commencing IFNβ treatment, with four out of six models achieving F1 score > 0.73, specificity > 0.92, and classification accuracy > 0.84. The decision tree achieved the most correct predictions at baseline. Although they are the easiest to interpret, decision trees are prone to overfitting and tend to be unstable. Consequently, we conclude the logistic regression models are the best choice for the classification of both classes (84% CA), whereas SVM is the best choice for identifying negative cases (98% specificity). We suggest that an ensemble model, combining several ML approaches, is more likely to prove optimal. In this way, models which were better at predicting positive cases could be combined with the models better at predicting negative cases to achieve superior performance. Clinically acceptable thresholds for model performance must be carefully considered, based on the medical, psychological and financial implications of incorrect predictions. In many cases existing tests using conventional biomedical techniques can be used as a benchmark. However, there is currently no method to predict ADA against IFNβ before starting treatment. It could be beneficial to investigate how MS patients feel about the risks of an incorrect result in the context of ADA prediction, and false positives or false negatives could be penalized accordingly.

We also produced models based on metabolite concentrations at M3 as IFNβ activates the immune system and effects systemic lipid levels (63, 64). Both the immune and metabolic responses to IFNβ treatment could influence the probability of ADA development. Model performance was comparable between M0 and M3, with the LR, LR+I, SVM and RF all achieving F1 score > 0.74, specificity > 0.84, and classification accuracy > 0.82. Overall the LR models had the most correct predictions at this timepoint. However, the contributing metabolites were dissimilar. This is unsurprising, as IFNβ had widespread effects on metabolite concentrations, which were likely to overwrite baseline differences. Interestingly, IFNβ inhibited a number of metabolites in ADA+ patients, suggesting a difference in IFN response. However, we cannot currently decipher to what extent the differences in IFN-response are truly related to ADA development, to patient intrinsic factors, or to the unequal distribution of treatment types between classes.

A limitation of our analysis was that our cohort received different types of IFNβ, which had different probabilities of inducing ADA development. Our sample size was insufficient to perform a comparison of only ADA– and ADA+ patients who were exposed to the same treatment. Indeed, when we examined the predictive metabolites at M3 several were only differentially expressed in patients on a particular IFNβ type. Therefore, any future validation of this work should be performed on a per treatment basis.

Notably, in this study nobody treated with intra-muscular IFNβ-1a (Avonex) developed ADA. Despite this, one patient treated with Avonex was predicted to be ADA+ by SVM, RF and the decision tree at M3. This suggests that the metabolic profile outweighed the type of treatment in this case. Thus, although the type of IFN was an important factor, the serum metabolites added an additional layer of personalized information. In addition to making predictions, the differences in metabolite concentrations and relationships identified here could be involved in the mechanisms driving ADA production. Excess cholesterol in the membrane leads to enhanced pro-inflammatory signaling in both macrophages (65, 66) and T cells (67), and we provided preliminary evidence that plasma membrane cholesterol is elevated in T cells isolated from ADA+ patients. Many of the lipids measured could influence T-cell cholesterol levels, including M-HDL-TG which featured in all of the models generated at baseline. Elevated triglyceride content of HDL is associated with its dysfunction and reduced capacity to support cholesterol efflux (68, 69). Therefore, the increased concentration of M-HDL-TG in patients who later became ADA+ could lead to abnormal cholesterol transport, and a predisposition to a pro-inflammatory immune response.

From a therapeutic perspective, it is possible that combining IFNβ treatment with an intervention to modify specific metabolites could protect against ADA development. In terms of lipid modification, there have already been several clinical trials comparing combination therapy of IFNβ with statins to IFNβ alone (70), although only one reported on the incidence of neutralizing antibodies and found no difference (71). It is important to note that the sample size was limited (*n* = 27), and statins may not be the most relevant therapeutic agents to modify the concentrations of the metabolites identified to be different in our analysis (e.g., HDL-TG). The metabolite networks revealed predictive metabolites that were highly interconnected which could be candidates for a widespread intervention, as well as unconnected metabolites which could be specifically targeted. Interactions between metabolites and patient characteristics were also identified– including baseline EDSS and acetyl acetate, smoking status and valine, and XXL-VLDL-TG with treatment type. If verified, these relationships could improve the personalization of treatment recommendations.

In conclusion, we have demonstrated the potential utility of serum metabolites and ML to predict the development of immunogenicity in MS patients. We suggest that the integration of additional molecular information (e.g., transcriptomics, genomics, proteomics) would strengthen these models, and provide novel insight into the interplay between lipids and the immunogenic response.

## Data Availability Statement

The metabolomics data presented in this study is available in Mendeley Data repository (72).

## Ethics Statement

The studies involving human participants were reviewed and approved by Ethics committee of the University College London Hospitals National Health Service Trust, London, United Kingdom (18/SC/0323 and 15/SW/0109) Medical Ethics Committee of the General University Hospital in Prague (125/12, Evropský grant 1.LF UK-CAGEKID) Ethikkommission der Fakultät für Medizin der Technischen Universität München, München, Germany (project no. 335/13) Ethikkommission Nordwest- und Zentralschweiz, Basel, Switzerland (project no. 305/13) Ethikkommission der Medizinischen Universität Innsbruck, Innsbruck, Austria (UN2013-0040_LEK). The patients/participants provided their written informed consent to participate in this study.

## Author Contributions

EJ, KW, MA, IP-T, and PD designed the research study. LC and AP performed ML analyses. PD and KW performed other statistical analyses. The manuscript was written by KW and EJ. AF-H coordinated anti-drug antibody testing. PN, EK, and RF provided patient serum samples and clinical assessment. All authors reviewed the manuscript and approved the final version.

## Conflict of Interest

AF-H has received speaker's fees from Pfizer, Biogen, Merck-Serono, and Sanofi-Genzyme. She has received unrestricted research support from, Biogen Idec and Pfizer. PN has received speaker honoraria from Biogen, Novartis, Merck, Roche and has been supported by Ministry of Education of the Czech Republic, project PROGRES Q27/LF1. EK received speaker honoraria and consultant fees from Actelion, Biogen, Celgene, Merck, Novartis, Roche, Sanofi and Teva, and support for research activities from Czech Ministry of Education [project Progres Q27/LF1]. RF has received honoraria and hospitality from Merck, Canbex pharmaceuticals Ltd, TEVA, Novartis, Genzyme, Allergan, Merz and Biogen. RF's current research activity is supported by the NIHR Biomedical Research Centre UCLH. PD was employed by SciCross AB. The remaining authors declare that the research was conducted in the absence of any commercial or financial relationships that could be construed as a potential conflict of interest.
