# Supplementary material for: Using Serum Metabolomics to Predict Development of Anti-drug Antibodies in Multiple Sclerosis Patients Treated With IFNβ
Source: Front Immunol. 2020 Jul 17;11:1527. doi: 10.3389/fimmu.2020.01527 (PMC7380268; doi:10.3389/fimmu.2020.01527)
Supplement: Supplementary file 4 [file Data_Sheet_4.pdf]

# *Supplementary Material*

## 1 Supplementary Data

**Supplementary File 1.** Excel spreadsheet containing the correlation matrix used for homology reduction.

**Supplementary File 2.** Lists of predictors for the support vector machine (SVM), random forest (RF), and logistic regression (LR) models at both month 0 (M0) ad month 3 (M3). These lists include both metabolic and clinical/demographic predictors. For LR models negative beta-coefficients indicate a reduced likelihood of ADA development.

**Supplementary File 3.** Excel spreadsheet containing the results of logistic regressions for individual metabolites, accompanying Supplementary Figure 1. P-values <0.05 are given in red text.

## 2 Supplementary Figures and Tables

**Supplementary Figure 1.** The association between each metabolite and ADA status was assessed by logistic regression adjusted for sex, age, BMI, smoking status, treatment type and dose, EDSS, and country of sample origin. Standard deviation-scaled odds ratios ± 95% confidence intervals are displayed as a forest plot. See also Supplementary File 3. Statistically significant associations ($p<0.05$) are represented by filled circles, and do not cross the central line. Metabolites shifted to the left are higher in ADA-, and conversely those shifted to the right are enriched in ADA+.

**Supplementary Table 1.** Full list of 158 metabolites used for analysis, including full names and abbreviations used throughout the main text. VLDL – very low density lipoprotein, IDL – intermediate density lipoprotein, LDL – low density lipoprotein and HDL – high density lipoprotein.

| Abbreviation | Full description |
|---|---|
| XXL-VLDL-P | Concentration of chylomicrons and extremely large VLDL particles |
| XXL-VLDL-L | Total lipids in chylomicrons and extremely large VLDL particles |
| XXL-VLDL-PL | Phospholipids in chylomicrons and extremely large VLDL particles |
| XXL-VLDL-C | Total cholesterol in chylomicrons and extremely large VLDL particles |
| XXL-VLDL-CE | Cholesterol esters in chylomicrons and extremely large VLDL particles |
| XXL-VLDL-FC | Free cholesterol in chylomicrons and extremely large VLDL particles |
| XXL-VLDL-TG | Triglycerides in chylomicrons and extremely large VLDL particles |
| XL-VLDL-P | Concentration of very large VLDL particles |
| XL-VLDL-L | Total lipids in very large VLDL particles |
| XL-VLDL-PL | Phospholipids in very large VLDL particles |
| XL-VLDL-C | Total cholesterol in very large VLDL particles |
| XL-VLDL-CE | Cholesterol esters in very large VLDL particles |
| XL-VLDL-FC | Free cholesterol in very large VLDL particles |

| | |
|---|---|
| XL-VLDL-TG | Triglycerides in very large VLDL particles |
| L-VLDL-P | Concentration of large VLDL particles |
| L-VLDL-L | Total lipids in large VLDL particles |
| L-VLDL-PL | Phospholipids in large VLDL particles |
| L-VLDL-C | Total cholesterol in large VLDL particles |
| L-VLDL-CE | Cholesterol esters in large VLDL particles |
| L-VLDL-FC | Free cholesterol in large VLDL particles |
| L-VLDL-TG | Triglycerides in large VLDL particles |
| M-VLDL-P | Concentration of medium VLDL particles |
| M-VLDL-L | Total lipids in medium VLDL particles |
| M-VLDL-PL | Phospholipids in medium VLDL particles |
| M-VLDL-C | Total cholesterol in medium VLDL particles |
| M-VLDL-CE | Cholesterol esters in medium VLDL particles |
| M-VLDL-FC | Free cholesterol in medium VLDL particles |
| M-VLDL-TG | Triglycerides in medium VLDL particles |
| S-VLDL-P | Concentration of small VLDL particles |
| S-VLDL-L | Total lipids in small VLDL particles |
| S-VLDL-PL | Phospholipids in small VLDL particles |
| S-VLDL-C | Total cholesterol in small VLDL particles |
| S-VLDL-CE | Cholesterol esters in small VLDL particles |
| S-VLDL-FC | Free cholesterol in small VLDL particles |
| S-VLDL-TG | Triglycerides in small VLDL particles |
| XS-VLDL-P | Concentration of very small VLDL particles |
| XS-VLDL-L | Total lipids in very small VLDL particles |
| XS-VLDL-PL | Phospholipids in very small VLDL particles |
| XS-VLDL-C | Total cholesterol in very small VLDL particles |
| XS-VLDL-CE | Cholesterol esters in very small VLDL particles |
| XS-VLDL-FC | Free cholesterol in very small VLDL particles |
| XS-VLDL-TG | Triglycerides in very small VLDL particles |
| IDL-P | Concentration of IDL particles |
| IDL-L | Total lipids in IDL particles |
| IDL-PL | Phospholipids in IDL particles |
| IDL-C | Total cholesterol in IDL particles |
| IDL-CE | Cholesterol esters in IDL particles |
| IDL-FC | Free cholesterol in IDL particles |
| IDL-TG | Triglycerides in IDL particles |
| L-LDL-P | Concentration of large LDL particles |
| L-LDL-L | Total lipids in large LDL particles |
| L-LDL-PL | Phospholipids in large LDL particles |
| L-LDL-C | Total cholesterol in large LDL particles |
| L-LDL-CE | Cholesterol esters in large LDL particles |
| L-LDL-FC | Free cholesterol in large LDL particles |
| L-LDL-TG | Triglycerides in large LDL particles |

| | |
|---|---|
| M-LDL-P | Concentration of medium LDL particles |
| M-LDL-L | Total lipids in medium LDL particles |
| M-LDL-PL | Phospholipids in medium LDL particles |
| M-LDL-C | Total cholesterol in medium LDL particles |
| M-LDL-CE | Cholesterol esters in medium LDL particles |
| M-LDL-FC | Free cholesterol in medium LDL particles |
| M-LDL-TG | Triglycerides in medium LDL particles |
| S-LDL-P | Concentration of small LDL particles |
| S-LDL-L | Total lipids in small LDL particles |
| S-LDL-PL | Phospholipids in small LDL particles |
| S-LDL-C | Total cholesterol in small LDL particles |
| S-LDL-CE | Cholesterol esters in small LDL particles |
| S-LDL-FC | Free cholesterol in small LDL particles |
| S-LDL-TG | Triglycerides in small LDL particles |
| XL-HDL-P | Concentration of very large HDL particles |
| XL-HDL-L | Total lipids in very large HDL particles |
| XL-HDL-PL | Phospholipids in very large HDL particles |
| XL-HDL-C | Total cholesterol in very large HDL particles |
| XL-HDL-CE | Cholesterol esters in very large HDL particles |
| XL-HDL-FC | Free cholesterol in very large HDL particles |
| XL-HDL-TG | Triglycerides in very large HDL particles |
| L-HDL-P | Concentration of large HDL particles |
| L-HDL-L | Total lipids in large HDL particles |
| L-HDL-PL | Phospholipids in large HDL particles |
| L-HDL-C | Total cholesterol in large HDL particles |
| L-HDL-CE | Cholesterol esters in large HDL particles |
| L-HDL-FC | Free cholesterol in large HDL particles |
| L-HDL-TG | Triglycerides in large HDL particles |
| M-HDL-P | Concentration of medium HDL particles |
| M-HDL-L | Total lipids in medium HDL particles |
| M-HDL-PL | Phospholipids in medium HDL particles |
| M-HDL-C | Total cholesterol in medium HDL particles |
| M-HDL-CE | Cholesterol esters in medium HDL particles |
| M-HDL-FC | Free cholesterol in medium HDL particles |
| M-HDL-TG | Triglycerides in medium HDL particles |
| S-HDL-P | Concentration of small HDL particles |
| S-HDL-L | Total lipids in small HDL particles |
| S-HDL-PL | Phospholipids in small HDL particles |
| S-HDL-C | Total cholesterol in small HDL particles |
| S-HDL-CE | Cholesterol esters in small HDL particles |
| S-HDL-FC | Free cholesterol in small HDL particles |
| S-HDL-TG | Triglycerides in small HDL particles |
| VLDL-D | Mean diameter for VLDL particles |
| LDL-D | Mean diameter for LDL particles |

| | |
|---|---|
| HDL-D | Mean diameter for HDL particles |
| Serum-C | Serum total cholesterol |
| VLDL-C | Total cholesterol in VLDL |
| Remnant-C | Remnant cholesterol (non-HDL, non-LDL cholesterol) |
| LDL-C | Total cholesterol in LDL |
| HDL-C | Total cholesterol in HDL |
| HDL2-C | Total cholesterol in HDL2 |
| HDL3-C | Total cholesterol in HDL3 |
| EstC | Esterified cholesterol |
| FreeC | Free cholesterol |
| Serum-TG | Serum total triglycerides |
| VLDL-TG | Triglycerides in VLDL |
| LDL-TG | Triglycerides in LDL |
| HDL-TG | Triglycerides in HDL |
| TotPG | Total phosphoglycerides |
| TG/PG | Ratio of triglycerides to phosphoglycerides |
| PC | Phosphatidylcholine and other cholines |
| SM | Sphingomyelins |
| TotCho | Total cholines |
| ApoA1 | Apolipoprotein A-I |
| ApoB | Apolipoprotein B |
| ApoB/ApoA1 | Ratio of apolipoprotein B to apolipoprotein A-I |
| TotFA | Total fatty acids |
| UnSat | Estimated degree of unsaturation |
| DHA | 22:6, docosahexaenoic acid |
| LA | 18:2, linoleic acid |
| FAw3 | Omega-3 fatty acids |
| FAw6 | Omega-6 fatty acids |
| PUFA | Polyunsaturated fatty acids |
| MUFA | Monounsaturated fatty acids |
| SFA | Saturated fatty acids |
| DHA/FA | Ratio of 22:6, docosahexaenoic acids to total fatty acids |
| LA/FA | Ratio of 18:2, linoleic acids to total fatty acids |
| FAw3/FA | Ratio of omega-3 fatty acids to total fatty acids |
| FAw6/FA | Ratio of omega-6 fatty acids to total fatty acids |
| PUFA/FA | Ratio of polyunsaturated fatty acids to total fatty acids |
| MUFA/FA | Ratio of monounsaturated fatty acids to total fatty acids |
| SFA/FA | Ratio of saturated fatty acids to total fatty acids |
| Glc | Glucose |
| Lac | Lactate |
| Pyr | Pyruvate |
| Cit | Citrate |
| Ala | Alanine |

| | |
|---|---|
| Gly | Glycine |
| His | Histidine |
| Ile | Isoleucine |
| Leu | Leucine |
| Val | Valine |
| Phe | Phenylalanine |
| Tyr | Tyrosine |
| Ace | Acetate |
| AcAce | Acetoacetate |
| bOHBut | 3-hydroxybutyrate |
| Crea | Creatinine |
| Alb | Albumin |
| Gp | Glycoprotein acetylation |

**Supplementary Table 2.** Lists of metabolites removed by homology reduction which are highly correlated to predictive markers contributing to models at M0 or M3. IDL – intermediate density lipoprotein, HDL – high density lipoprotein, LDL – low density lipoprotein, VLDL – very low density lipoprotein, (XX)S – (extra extra) small, M – medium, (XX)L – (extra extra) large, -C – cholesterol, -CE – cholesterol esters, -FC- free cholesterol, -L – lipids, -P – particle concentration, -PL – phospholipids, -TG – triglycerides.

**M0**

| Metabolite | Full name | Correlated features |
|---|---|---|
| **ApoB/ApoA1** | Apolipoprotein B/A1 ratio | - |
| **M-HDL-TG** | Triglycerides in medium HDL | - |
| **M-VLDL-CE** | Cholesterol esters in medium VLDL | VLDL-C<br>M-VLDL-C<br>S-VLDL-L, -P |
| **Remnant-C** | Remnant cholesterol | ApoB |
| **TG/PG** | Triglyceride/phosphor-glyceride ratio | VLDL-TG<br>L-VLDL-C, -L, -P, -TG<br>M-VLDL-FC, -L, -P, -PL,-TG<br>S-VLDL-TG |
| **VLDL-D** | VLDL diameter | - |
| **XXL-VLDL-FC** | Free cholesterol in extra extra large VLDL | Serum-TG, VLDL-TG<br>XXL-VLDL -C, -L, -P, -PL, -TG,<br>XL-VLDL-C, -CE, -FC, -L, -P, -PL, -TG<br>L-VLDL-C, -FC, -L, -P, -PL, -TG<br>M-VLDL-FC |

**M3**

| Metabolite | Full name | Correlated features |
|---|---|---|
| **AcAce** | Acetyl-acetate | - |
| **Glc** | Glucose | - |
| **MUFA/FA** | Monounsaturated fatty acid/fatty acid ratio | - |
| **XXL-VLDL-FC** | Free cholesterol in extra extra large VLDL | L-VLDL-C, -FC, -L, -P, -PL, -TG,<br>XL-VLDL-C, -CE, -FC, -L, -P, -PL, -TG,<br>XXL-VLDL-C, -L, -P, -PL, -TG |

**Supplementary Table 3.** Comparison of model predictions for each sample at baseline (M0) or 3 months (M3). Predicted ADA- (-) or predicted ADA+ (+). Number of different prediction is summarized for each class, and total cohort.

| | M0 Model Predictions | | | | | M3 Model Predictions | | | | |
|---|---|---|---|---|---|---|---|---|---|---|
| | Tree | SVM | LR | LR + I | RF | Tree | SVM | LR | LR + I | RF |
| ADA- | | | | | | | | | | |
| 1 | - | - | - | - | - | - | - | - | - | - |
| 8 | - | - | - | - | - | + | + | - | - | + |
| 9 | - | - | - | - | - | - | - | - | - | - |
| 10 | - | - | - | - | - | - | - | - | - | - |
| 11 | + | - | + | + | - | - | - | + | + | + |
| 12 | - | - | + | + | - | + | + | + | + | + |
| 13 | - | - | - | - | - | - | - | - | - | - |
| 14 | - | - | - | - | - | - | - | - | - | - |
| 15 | - | - | - | - | - | - | - | - | - | - |
| 16 | - | - | - | - | - | - | - | - | - | - |
| 17 | - | - | - | - | - | - | - | - | - | - |
| 18 | - | - | - | - | - | - | - | - | - | - |
| 19 | - | - | - | - | - | - | - | - | - | - |
| 20 | - | - | - | - | - | - | + | - | - | - |
| 21 | - | - | - | - | + | - | - | - | - | - |
| 22 | - | - | - | - | - | - | - | - | - | - |
| 23 | - | - | - | - | + | - | - | - | - | - |
| 24 | - | - | - | - | - | - | - | - | - | - |
| 25 | - | - | - | - | - | - | - | - | - | - |
| 26 | - | - | - | - | - | - | - | - | - | - |
| 27 | - | - | - | - | - | - | - | - | - | - |
| 28 | - | - | - | - | - | + | - | - | - | + |
| 29 | - | - | - | - | - | - | - | - | - | - |
| 30 | - | - | - | - | - | - | - | - | - | - |
| 31 | - | - | - | - | - | - | - | - | - | - |
| 32 | - | - | - | - | - | - | - | - | - | - |
| 33 | - | - | - | - | - | - | - | - | - | - |
| 34 | - | - | - | - | + | + | - | - | - | + |
| 35 | - | - | - | - | - | - | - | - | - | - |
| 36 | - | - | - | - | - | - | - | - | - | - |
| 37 | - | - | - | - | + | - | - | - | - | - |
| 38 | - | - | - | - | - | - | - | - | - | - |
| 39 | - | - | - | - | - | - | - | - | - | - |
| 40 | - | - | - | - | - | - | - | - | - | - |
| 41 | - | - | - | - | + | - | - | - | - | - |
| 42 | + | + | + | + | + | + | + | + | + | + |
| 43 | - | - | - | - | - | - | - | - | - | - |
| 44 | - | - | - | - | - | - | - | - | - | - |
| 45 | - | - | - | - | + | - | - | - | - | - |
| 46 | - | - | - | - | - | + | - | - | - | - |
| 47 | - | - | - | - | - | - | - | - | - | - |
| 48 | - | - | - | - | - | - | - | - | - | - |
| 49 | - | - | - | - | - | - | - | - | - | - |
| 50 | - | - | - | - | - | - | - | - | - | - |
| 51 | + | - | + | + | - | + | + | + | + | + |

| | | | | | | | | | | |
|---|---|---|---|---|---|---|---|---|---|---|
| 52 | - | - | - | - | - | - | - | - | - | - |
| | | | ADA- discordant predictions | | | 6 | 4 | 0 | 0 | 9 |
| ADA+ | | | | | | | | | | |
| 53 | + | + | + | + | + | + | + | + | + | + |
| 54 | + | - | - | - | + | + | - | + | + | + |
| 58 | + | + | + | + | + | + | + | + | + | + |
| 59 | + | - | + | + | - | + | + | + | + | + |
| 60 | + | + | + | + | + | + | + | + | + | + |
| 61 | + | + | + | + | + | + | + | + | + | + |
| 62 | + | - | + | + | + | + | + | + | + | + |
| 63 | + | + | + | + | + | + | + | + | + | + |
| 64 | + | + | + | + | + | + | - | + | + | + |
| 65 | + | + | + | + | + | + | + | + | + | + |
| 66 | - | - | - | - | + | - | + | - | - | + |
| 67 | - | - | - | - | - | - | - | - | - | - |
| 68 | - | + | + | + | + | - | + | + | + | + |
| 69 | + | + | + | + | + | + | + | + | + | + |
| 70 | - | + | + | + | + | + | + | + | + | + |
| 71 | - | + | + | + | + | + | + | + | + | + |
| 72 | + | + | + | + | + | + | + | + | + | + |
| 73 | + | - | - | - | - | - | - | - | - | - |
| 74 | + | + | + | + | + | + | + | + | + | + |
| 75 | + | - | - | - | - | - | - | - | - | - |
| 76 | - | + | + | + | + | + | + | + | + | + |
| 77 | + | + | - | - | - | - | - | - | - | - |
| 78 | + | + | + | + | - | + | + | + | + | + |
| 79 | + | - | + | + | - | + | - | + | + | - |
| 80 | - | - | + | + | - | + | + | + | + | + |
| 81 | + | + | + | + | + | + | + | + | + | + |
| 82 | + | - | - | - | - | - | - | - | - | - |
| | | | ADA+ discordant predictions | | | 8 | 6 | 1 | 1 | 2 |
| | | | **Total discordant predictions** | | | **14** | **10** | **1** | **1** | **11** |
