# Supplementary figures and images for: Using Serum Metabolomics to Predict Development of Anti-drug Antibodies in Multiple Sclerosis Patients Treated With IFNβ
Source: Front Immunol. 2020 Jul 17;11:1527. doi: 10.3389/fimmu.2020.01527 (PMC7380268; doi:10.3389/fimmu.2020.01527)

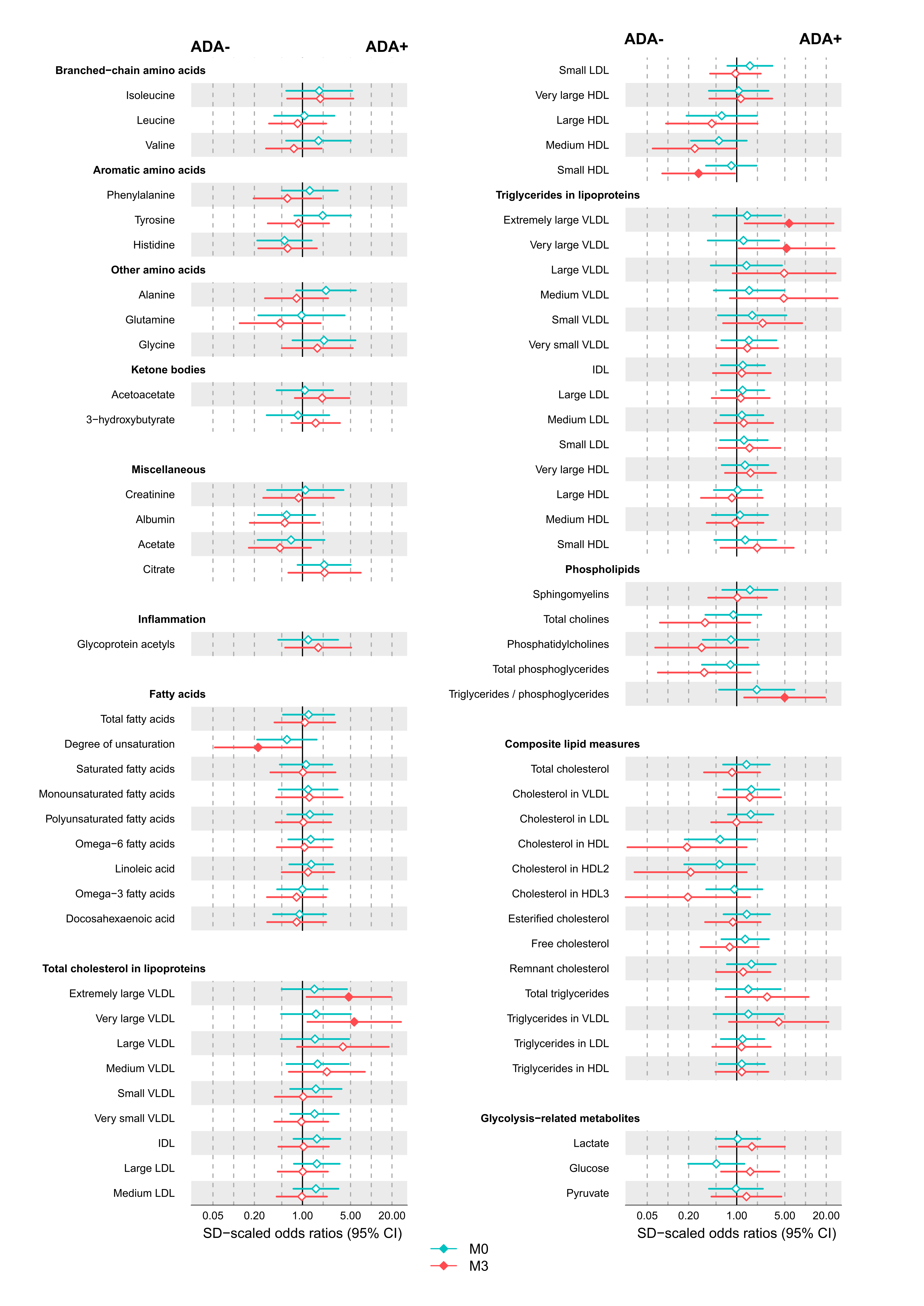

Supplement: Supplementary file 5 [file Image_1.JPEG]

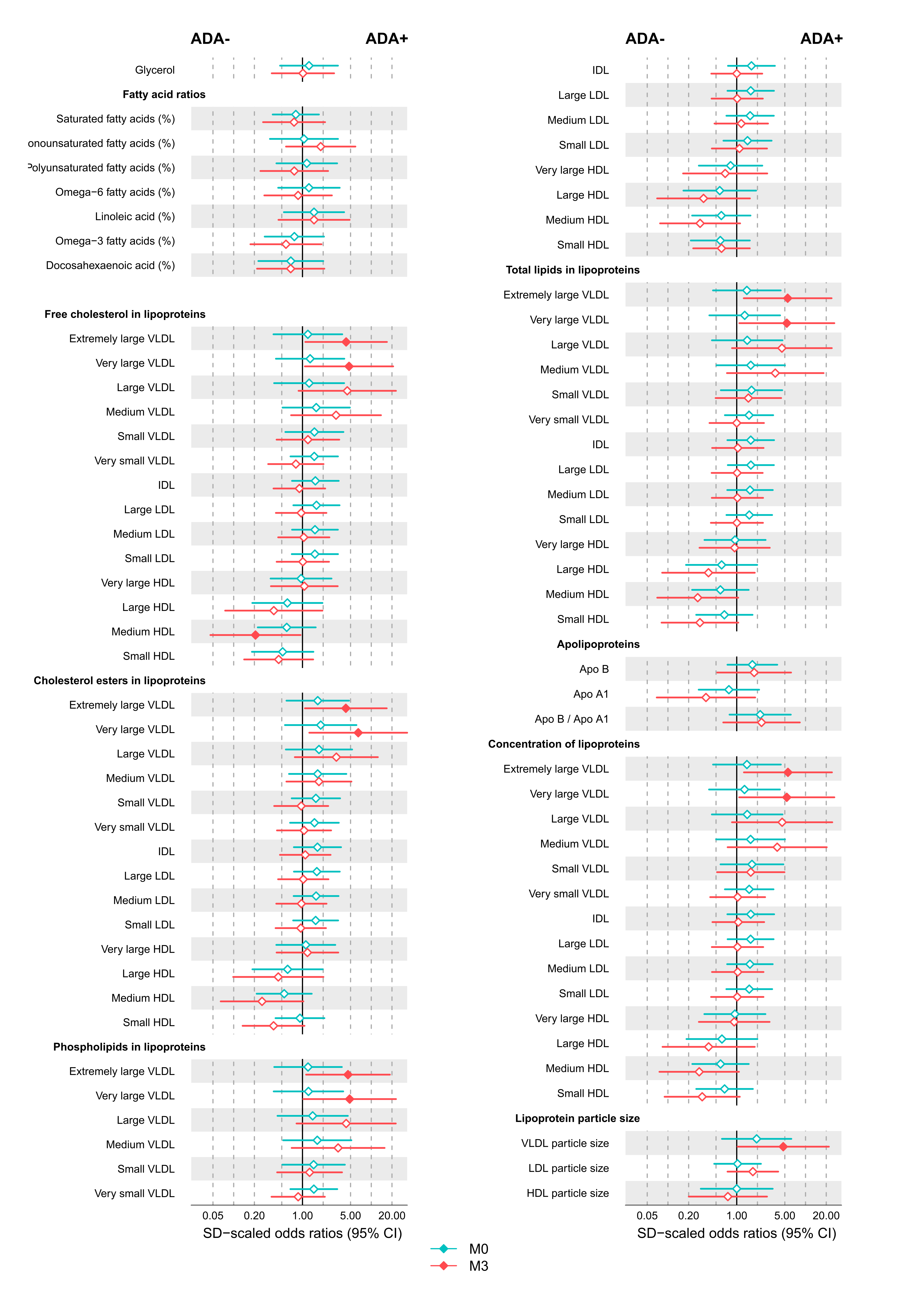

Supplement: Supplementary file 6 [file Image_2.JPEG]
